# Is It the Beginning of the End?

**Published:** 1895-02

**Authors:** James Weir

**Affiliations:** Owensboro, Ky.


### Is it the Beginning of the End?

BY JAMES WEIR, JR., M. D., OWENSBORO, KY.

When we come to examine the history of the world we find evidence that certain nations have, at times, reached a high state of prosperity, and have then degenerated to such a degree that they have either passed entirely out of existence, or have lapsed into a state of semi-barbarity. This has generally been brought about by conquest, but the races conquered had first become enfeebled by their habitudes of thought and manner of living. It is a well-established fact that luxury brings debauchery, and that debauchery occasions degeneration. All nations that have, heretofore, reached the zenith of their prosperity, have been engulfed, at some time or other, in the maelstrom of luxurious habits, and haven fallen under the lethal influence of a degeneration occasioned solely by debauchery; for the luxury and debauchery of one class brought increased poverty on, as well as excess in, other classes, and poverty and excess are prominent factors in the production of degeneration, as we shall see further on in this paper. Says the brilliant author of "Psychopathia Sexualis," Krafft-Ebing: "Periods of moral decadence in the life of a people are always contemporaneous with times of effeminacy, sensuality, and luxury. These conditions can only be conceived as occurring with increased demands upon the nervous system, which must meet these requirements. As a result of increase of nervousness, there is increase of sensuality, and, since this leads to excesses among the masses, it undermines the foundations of society—the morality and purity of family life. When this is destroyed by excesses, unfaithfulness, and luxury, then the destruction of the state is inevitably compassed in material, moral, and political ruin."

Such was the condition of the Latin race when the fierce and hardy Vandals overran the Roman peninsula; such was the condition of the Assyrians when Babylon fell beneath the onslaughts of the great Macedonian; such was the condition of the Egyptians when the northern myriads swept down upon the fertile valley of the Nile and destroyed forever the once powerful and all conquering kingdom of the Pharaohs; and such, too, was the condition of the French nation in 1794, when anarchy unfurled its red banner at the head of the most gigantic social revolution the world has ever known. At the present time, community of interests, as well as higher civilization, would utterly forbid the total subjugation of one civilized nation by another, such as occurred in the olden times; hence no nation need fear annihilation from such a source. The danger comes from another point, and consists in the almost certain uprising, at some time in the future, of degenerate individuals in open warfare and rebellion against society.

The question whether the world is growing better or worse is often debated, and can be answered affirmatively on both sides. Better, because superstition, bigotry, and dogmatism have given away, to a great extent, to the tolerance and freedom of higher civilization and purer ethics in normal, healthy man; worse, because crime (and I mean by crime *all* antisocial acts) has greatly increased on account of the pernicious influence of degeneration. That supersitition, bigotry, and dogmatism are on the wane, and that they will, sooner or later be entombed in that depository of obsolete savage mental hebetude—absolute and utter oblivion—a glance at the success that science has achieved in the warfare waged against it by the church, will at once declare. (Throughout this article I use the word church to express priests of any and every denomination, whether Jew, Gentile, or Pagan, Protestant or Catholic.) A short incursion into this subject, i. e., the church's warfare on science, is absolutely necessary, for the triumph of science over its enemies—superstition, bigotry, and dogmatism, coincidently, ignorance and illiterateness—shows that the civilized world, at the present time, is markedly different in some respects from the world of ancient, mediæval, and even comparatively recent times; and, in summing up, this changed condition will be a weighty factor in making up an answer to the question which heads this paper.

When Olympus first faded away from the enlightened eyesight of the Greeks, and changed into space besprinkled with stars; when Zeus no longer held his divine court on its mystic summit; when oracles became mute and the fabled wonders of the "Odyssey" either vanished or resolved themselves into prosaic commonplaces under the investigations of the sceptic or the accidental discoverer, the church made a most strenuous protest against the destruction of its traditions. Some of these early seekers after truth were killed and their goods confiscated. The church issued its edict against heresy (and any doctrine that taught a belief antagonistic to the accepted tenets of pagan mythology and theogony was heresy), and hurled its anathemas against the heretic. Olympus, in the eyes of the church, still existed, and Zeus, the man-god, still quaffed the sacred ambrosia in its shady groves. The Sirens still sang their entrancing songs, while Scylla and Charybdis were ever stretching out eager arms toward unwary mariners. Gigantic one-eyed Cyclops, with Polyphemus as their leader, still patrolled the shores of Sicily and kept their "ever-watchful eyes" turned toward the open sea. The hardy Greek sailor landed on the Cyclopean island, and discovered that Polyphemus, and Argus, and Brontes, and Steropes, and all the other one-eyed monsters were nothing but sea-wrack, boulders and weeds. He sailed farther, past Scylla and Charybdis, and discovered no greater dangers than sharp rocks and whirlpools. Yet farther he sailed out into the mysterious sea, and the only Siren's song he heard was the whistling of the wind through the cordage of his vessel. In vain the church thundered against the daring investigator. Neither fire, nor sword, nor imprisonment, nor death itself, could check the march of truth. Mythology and pagan theogony had received their death-blows; superstition, bigotry, and dogmatism were elbowed aside and gave place to dawning science. The church held that that which had been believed by pious men for untold ages must necessarily be true. Science, in the garb of philosophy, with cold, dispassionate criticism proved that these hitherto accepted truths were arrant fallacies. The poets and writers then took up the subject, and finally the people fell into line; so superstitious, bigoted, dogmatic mythology died, intellectuality took its place, and higher civilization took a step forward.

With every new discovery, with every victory over superstition, bigotry, and dogmatism, civilization took a step upward until it stands to-day as far above the civilization of those old days as do the giant stems of the mighty red-woods above the chaparal and undergrowth of the California forests. In its battle with superstition civilization has grown strong, hardy, and, above all, vigilant. This last quality it will need most of all in its coming battle with the combined hosts of antisocial degenerates. In its battle against the church, civilization has gained the right to think for itself. It has demanded, and is now receiving, to some extent, the right of education, of erudition; and education is, and will be, a most potent warrior against degeneration.

That a luxurious manner of living eventually leads to debauchery, and that debauchery is a prime factor in creating degeneration, no physiologist of the present day will for one instant deny. I wish to show, in this paper, that luxury is hurrying us toward a social cataclysm, beside which the downfall of the Roman Empire, the destruction of ancient Egyptian and Babylonian civilizations, and the bloody days of the French Revolution, will sink into utter insignificance.

A brief *resume* of certain historical epochs will be necessary in order to furnish a parallel from which I wish to draw several indisputable and incontrovertible conclusions.

The Roman people, under the leadership of its ancient heroes, was a nation of hardy warriors and husbandmen. That preeminent military genius, Julius Cæsar, had carefully fostered this warlike spirit in the bosoms of his compatriots, and by a series of brilliant campaigns had made the Roman nation the most powerful on the face of the globe. The Roman legions were not only victorious on land, extending their conquests into Iberia, farther Gaul, and still farther Britain, but the Roman triremes also swept the Mediterranean, from the Pillars of Hercules to the shores of Syria and Egypt. Wealth poured into the country from all sides and the people revelled in a boundless prosperity. Luxury had already begun to enervate the hardy soldiery at the time of Cæsar's assassination, yet not enough to show degeneration and demoralization. The empire under the first emperors steadily grew richer and more powerful, and the luxury of the rich more unlimited and licentious. At length a change can be noticed. The Roman legions, hitherto victorious over every foe, are now frequently vanquished; conquered tribes upreared the standard of revolt and refuse to pay tribute; the territorial boundaries of the empire materially shrink, and its once conquered provinces pass out of its dominion forever. The gradual degeneration of this nation is faithfully mirrored in the characters of the emperors who governed it. Nero, Caligula, Tiberius, Caracalla, and Messalina, the depraved wife of Claudius and daughter of Domitia Lepida, herself a licentious and libidinous woman, were but accentuated types of the luxurious and debauched nobility. Not only did the nobility become victims of degeneration, but the poorer classes also lost their virility, until at last we find the stability of the nation preserved through the instrumentality of foreign mercenaries. The greatness of this once widespread empire dwindled away, the freedom of its institutions contracting along with its shrinking boundaries, until we find it lapsed into a state of barbarian despotism under the son of Aurelius; and, had it not been for outside influences, it would have eventually fallen into a state of utter and complete savagery.

Now, let us turn to a much older civilization. When the first conquerors of Egypt about whom history can tell us so little, first occupied the fertile valley of the Nile, the country, in all probability, was inhabited by negroes. This conquering race drove out or enslaved the native population and founded the ancient kingdom of Egypt. This kingdom waxed strong and mighty until at the time of Rameses the Great, three thousand two hundred years ago, it was the most powerful monarchy in the whole world. This mighty son of Ra, Meiamoun Ra, or Rameses, as he is most generally styled, was a warrior and a statesman. He led his victorious troops north, east, and west, conquering nations as he went, until he dominated and brought into a state of vassalage over two-thirds of the known world. Wealth flowed into his kingdom from all the surrounding countries, consequently luxury, with its never-failing associate, debauchery, made their appearance and the decadence of this mighty kingdom set in. It is true that many Pharaohs reigned after Rameses, and that the monarchy maintained its greatness for a long period of time; but luxury had taken hold on the people at the time of their greatest prosperity and had sown the seeds of degeneration, which flourished and grew apace, until the emasculated and effeminate people yielded up their independence to the conquerors, and passed out of existence as a nation forever.

Now, let us turn to a recent civilization. At the time of Louis XVI. the French nation was thorougbly under the influence of degeneration consequent to a luxury and licentiousness that had had a cumulative action for several hundred years. The peasantry and the inhabitants of the faubourgs, owing to their extreme poverty, itself a powerful factor in the production of degeneration, had lapsed into a psychical state closely akin to that of their savage ancestors. The nobility were weak and effeminate, the majority of them either sexual perverts, or monsters of sensuality and lechery. The middle class, then as ever the true conservators of society, seeing this miserable state of affairs, attempted to remedy it. Not fully understanding the dangers of such a procedure, they allowed the degenerate element to share in their deliberations. Their moderate and sensible counsels were quickly overruled by their savage associates, who brought about a Reign of Terror (with such psychical atavists as Marat, Danton, and Robespierre at its head), the like of which the world had never seen before nor has ever experienced since. I have demonstrated, in the three instances of history cited above, that degeneration has invariably followed luxury, and that a social and political revolution has been, invariably the result of this degeneration; therefore, as we ourselves are entering upon an epoch closely akin to the three several epochs just mentioned, it will be well for us to study the phenomena that bring about such revolutions.

It is conceded by everyone that man completed his cycle of physical evolution many thousands of years ago. Since his evolution from his pithecoid ancestor the forces of nature have been at work evolving man's psychical being. Now, man's psychical being is intimately connected with, and dependent on, his physical being, therefore it follows that degeneration of his physical organism will necessarily engender psychical degeneration. Hence, if I can prove that man, by leading a life of luxury or one of poverty and want, produces physical degeneration, it will naturally follow that psychical degeneration will also accrue; and, as one of the invariable results of degeneration is atavism or reversion, both physical and psychical, the phenomenon of a social revolution characterized by pronounced savagery and barbarity, in which society is overthrown and anarchy instituted in its stead, will no longer appear strange and unnatural.

Neurasthenia, or the loss of nervous tonicity, is a prime factor in the production of degeneration. The offspring of neurasthenic parents always show degeneration in some form or other. That luxury produces neurasthenia can be demonstrated beyond the shadow of a doubt. Nine-tenths of the clientele of the gynecologist is derived from the wealthy, luxurious and fashionable class. The same may be said of the neurologist and alienist. Paresis and kindred forms of insanity are, almost exclusively, forms of degeneration affecting wealthy people, while the proportion of sexual perverts among the rich is remarkably high.

Let us see if we cannot discover some of the factors in the causation of such wide-spread and abundant neuroses among those fashionable and luxurious individuals who arrogate to themselves the title of "Society." Man is, naturally, a diurnal animal, but the fashionable world has reversed the natural order of things and has made him a nocturnal animal. Now, the long continued influence of artificial light exerts a very deleterious effect on the nervous system; hence, it is not to be wondered at that so many men and women of society are neurasthenic. Not only are those individuals who, voluntarily and preferably, spend the greater portions of their lives in artificial light, rendered nervously irritable, but those also who are driven by force of circumstances to turn night into day are likewise afflicted. Several years ago I met a distinguished editor at Waukesha, who was suffering greatly from nervous exhaustion. He told me that he was so situated that he did all of his work at night, often writing until three o'clock in the morning. I advised him to quit this and to do his editorial work during daylight. Not long after he wrote me that he had followed my advice, and that he was a new man in point of health. The loss of nervous vitality makes itself evident by a feeling either of exhaustion or irritability. The fashionable devotee, in order to counteract this, either stimulates the system with alcohol, or exorcises the "fidgets" by the use of sedatives, such as chloral or morphia. The baneful effects of such medication are not at once appreciable, but if continued for any length of time they will eventually result in a total demoralization of the nervous system. Time and again have I seen fashionable men and women, at the close of the season, veritable nervous wrecks. What necessarily would be the effect of physical and psychical lesions like these on a child begotten by such parents? The inevitable result would be degeneration in some form or other. Again, many men and women stand the drain of a fashionable season on their nervous systems without attempting to recoup through the agency of drugs, and at the end find themselves physically and psychically exhausted. They go to the seaside or some other resort, and, in a measure, recover their nervous vitality, only lose it again during the next season. This continues for season after season, the nervous system all the time becoming weaker, until some day there is a collapse ending in hysteria, paresis, or some other of the hundred forms of neurotic disorder. What will be the effect on the progeny resulting from the union of such individuals? Again the answer must necessarily be—degeneration. Artificial light is not the only cause of this nervous irritability. The long and continued intercourse of the sexes in the ball room, where the women are dressed so *decollete* that they excite sensuality in the men, very frequently without the men being conscious of the fact, must necessarily exert a deleterious effect on the nervous system. Contact of the sexes in the dance is only pleasurable because of that contact. I am fully aware of the fact that this idea is scouted and denied by those who indulge in the waltz and kindred dances. They claim that no thought of carnality ever enters into their feeling. I know from personal experience that they are honest in this declaration, yet from a psychical standpoint they are woefully in error. Æstheticism and carnality are by no means as dissociate as the æsthete would have us believe. All pleasurable emotions that have their inception in the senses are fundamentally of carnal origin. The waltz is æsthetic, yet all of its pleasure is based on an emotion closely akin to sensuality. Men derive no pleasure from waltzing with one another, nor do women under like circumstances.

Nature demands, in the interest of health, a certain amount of exercise. The luxurious society man or woman utterly disregards this demand of nature, consequently indigestion, with all of its associated ills steps in, and becomes an additional factor in the production of nervous exhaustion. To tempt the appetite, highly seasoned foods, many of which are deleterious and injurious, are prepared and taken into the torpid and crippled stomach. Finally, nature rebels, and the unfortunate dyspeptic is forced to go through life on a diet of oatmeal, or, weakened by lack of healthy sustenance, the brain gives way and the victim passes the remainder of his or her life in a lunatic asylum. Children begotten by miserable invalids like these, beyond a peradventure, must necessarily be degenerate. Indigestion is not the only ill that nature inflicts for any disregards of her laws. She is a rough nurse but a safe one, consequently she forbids the rearing of her hardiest creation, man, in hot houses, as though he were a tender exotic. The luxurious individual pampers his body, following the dictates of his own selfish desires and ntterly disregarding the laws of nature, and, before he reaches middle age, discovers that he has become an old, old man, weak in body, but still weaker in mind. The children resulting from the union of the various neurasthenics described above are necessarily degenerate. As they grow up they show this degeneration by engaging in all kinds of licentious debauchery, and unnatural and perverted indulgences of appetite. In nine cases out of ten they will spend the fortunes inherited from their parents in riotous debauchery, and will eventually sink, if death does not overtake them, to the level of their fellow-degenerates—those who have been brought into existence by poverty and debauchery, and who await them at the foot of the social ladder. Among such degenerate beings the doctrine of socialism, of communism, of nihilism, and of anarchy have their origin.

Now, let us turn our attention to the evidences of luxury and debauchery, and the consequent evidences of degeneration which obtrude themselves on all sides. The reckless extravagance of the nobility of the old world is well known. Vice and licentiousness even penetrate to the royal households, and princes of the blood pose as roués and debauchees. As I have demonstrated elsewhere, degeneration in the wealthy classes of society generally makes itself evident by the appearance of psycho-sexual disorders. The horrible abominations of the English nobility, as portrayed in the revelations of Mr. Stead, are well known. Charcot, Segalas, Féré, and Bouvier, give clear and succinct accounts of the vast amount of sexual perversion existing among the French, while Krafft-Ebing informs us that the German empire is cursed by the presence of thousands of these unfortunates. When we come to examine this phase of degeneration in our own country, we find that it is very prevalent. This is especially noticeable in the larger cities, though we find examples of it scattered broadcast throughout the land. The editor of one of our leading magazines, in a remarkable series of letters, has shown that the wealthy New Yorkers revel in a luxuriousness that is absolutely startling in its license. Thousands are expended on a single banquet, while the flower bills for a single year of some of these modern Luculli would support a family of five people for three or four years. Bacchanalian orgies that dim even those of the depraved, corrupt, and degenerate Nero, are of nightly occurrence. Drunkenness, lechery, and gambling, are the sports and pastimes of these ultra-rich men, and it is even whispered that milady is not much behind milord in the pursuit of forbidden pleasures.

Psycho-sexual disorders are not the only evidences of degeneration in the wealthy, by any means. Many a congenital criminal is born in the purple, who shows his moral imbecility in many ways. Sometimes he sinks at once to the level of a common thief, but generally his education keeps him within the pale of the law. Always, however, his sensuality is unbounded, and he will hesitate at nothing in order to gratify his desires. This unbridled license has already had its effect elsewhere. We see that it has even corrupted the guardians and conservators of the public peace. The recent investigation of the police board of New York shows a degree of corruption that is simply overwhelming, and that the same state of affairs exist in Chicago, New Orleans, St. Louis, and other large cities, I have every reason to believe. There are yet other evidences of degeneration; witness the eroticism that is to be found in our literature. Unless a book appeals to the degenerate tastes of its readers, it might just as well never have been published. This is not cynicism; it is plain, unvarnished truth—witness the success of "His Private Character," of "Is This Your Son, My Lord?" of hundreds of other works of the same character. Again, turn to the stage and we find the same thing. The tragedies and comedies of Shakespeare are shelved, while society plays and "living pictures" hold the boards. Salacity, with only sufficient covering to barely hide downright lewdness, is everywhere apparent. Now, what is the result of all this? There can be but one answer, and that is degeneration. That which happened centuries ago will happen again, for man is governed by the same laws of nature now as he was then.

Statistics show that insanity is markedly on the increase. This is not to be wondered at when we take into consideration the fact that debauchery is the rule, and not the exception, among certain classes of people. Syphilis, one of the most productive causes of degeneration, is exceedingly active throughout the whole civilized world. Blashko states that one out of every nine or ten men in the city of Berlin is tainted with syphilis. This is wholly attributable to the unbounded sensuality of the people. Crime of every description is rearing its hydrahead and clasping in its embrace an alarming proportion of human beings. I have shown elsewhere[1] that the congenital criminal is the result of degeneration, and that he comes from all classes of society. He is, however, most frequently the product of the lower class, and lives and dies among his congeners. I have shown also[2] that the anarchist, the nihilist, and the socialist, belong to the same category of degenerate beings. Poverty, brought on by the luxury of the rich, by war, and by high taxation, has, during the last millenary period, been very fertile in the production of degeneration in the old world. Lack of food and sanitation, the usual adjuncts of poverty, are powerful factors in the production of degenerate individuals. The old world has gotten rid of these people as rapidly as possible by unloading them on our shores. Year after year, practically without restriction, thousands of these antisocial men and women have swarmed into our country, until we, comparatively speaking, a nation just born, contain as many of these undesirable citizens as any of the older nations. They still continue to enter our gates, and we are adding to their number, as I have shown, by our own production. Some day—and I greatly fear that day is not very far distant—some professional anarchist (for there are professional anarchists as well as professional thieves) will consider that the time is ripe for rebellion, and, raising the fraudulent cry of "Labor against Capital," instead of his legitimate cry, which is "Rapine, Murder, Booty!" will lead this army of degenerates, composed of anarchists, socialists, nihilists, sexual perverts, and congenital criminals, against society. And who will bear the brunt of this savage onslaught? The ultra-rich? By no means. The great middle class—the true conservators of society and civilization, will fight this battle. It will be a strife between civilization and degeneration, and civilization will carry the day. There would have been no French Revolution had the middle class been as wise then as it is to-day. They were taken by surprise at that savage, bloody time, but as soon as they recovered, how quickly they brought order out of chaos! Education is the bulwark of civilization, and the great middle class, freed of dogmatism, bigotry, and supersti-

---

1 Vide American Naturalist, The Recidivist.

2 Vide Century Magazine for October, The Methods of the Rioting Striker an Evidence of Degeneration.

tion, is welcoming education with open arms. It is gaining recruits, and is strengthening its defenses, so that when the end comes its enemies may find it fully prepared. When this fight takes place, millions of dollars' worth of property will be burned, and thousands of lives will be sacrificed, but when the smoke of battle clears away, civilization will be declared the victor. And the ultra-rich, what of them? They will simply open their purses, like they did in ancient Rome, and pay for the privilege of being protected. The sober middle class is a business people, and they will demand and obtain assistance from their wealthy brethren. From the signs of the times and the evidence before me, I have no hesitation in declaring that I believe that the beginning of the end is at hand. This social cataclysm may not occur for many years, yet the agencies through which it will finally be evolved, are even now at work, and are bringing the culmination of their labors ever nearer and nearer as time passes.—*N. Y. Medical Record, Dec. 29, 1894.*

---

### Texas State Medical Association.

### SECTION ON OPHTHALMOLOGY AND OTOLOGY.

Physicians interested in this branch of practice, and desiring to contribute papers for the next meeting of the State Medical Association (4th Tuesday in April), are invited to do so, and are respectfully requested to notify me, giving title of paper, not later than April 10th. This is desirable and necessary, in order that a proper place in the proceedings may be assigned each paper, and the title, and hour set for reading paper, may appear in the programme. ROBERT E. MOSS, M.D., Chairman,

San Antonio, Texas.

---

PROF. LOOMIS died recently, as, we suppose, all medical men know by now. He left over a million dollars. Of this, he gave a hundred thousand to his son Harry, and a like amount to the New York Academy of Medicine, in trust, the interest to be used annually in giving an entertainment. Now, that's nice. They will meet, but they will miss him, and will drink champagne to his memory, and bless him.

